# To Act or Not to Act—a Sense of Control Is Important for People With Chronic Obstructive Pulmonary Disease to Increase Physical Activity: Grounded Theory Study

**DOI:** 10.2196/39969

**Published:** 2023-02-03

**Authors:** Sarah Marklund, Ann Sörlin, Tobias Stenlund, Karin Wadell, Andre Nyberg

**Affiliations:** 1 Section of Physiotherapy Department of Community Medicine and Rehabilitation Umeå University Umeå Sweden

**Keywords:** physical activity, chronic obstructive pulmonary disease, COPD, eHealth, interviews, eHealth tools

## Abstract

**Background:**

Among people with chronic obstructive pulmonary disease (COPD), low level of daily physical activity (PA) is the main risk factors for developing cardiovascular, metabolic, and musculoskeletal comorbidities. Increasing PA in people with COPD is complex as PA behavior itself is complex and multifaceted, including personal, physiological, and psychological elements as well as social and environmental factors. Although eHealth solutions such as web-based support or websites have shown positive effects on PA in people with COPD, the results are inconclusive, and it is still unclear how eHealth solutions might be used to support positive changes in PA behavior in people with COPD.

**Objective:**

This study aimed to explore the perceptions of increasing objective PA when using a web-based eHealth tool among people with COPD.

**Methods:**

This study was part of a pragmatic randomized controlled trial with in-depth interviews between the 3- and 12-month follow-ups. The methodology used was constructivist grounded theory. All sampling included participants from the randomized controlled trial intervention group, that is, participants who had access to the eHealth tool in question and agreed to be contacted for an in-depth interview. Inclusion of participants continued until data saturation was reached, resulting in an inclusion of 13 (n=7, 54% women) participants aged between 49 and 84 years and living in 8 municipalities in Middle and Northern Sweden. Two interviews were conducted face-to-face, and the remaining interviews were conducted via telephone. All interviews were recorded using a Dictaphone.

**Results:**

The analysis resulted in 3 main categories: welcoming or not welcoming action, having or lacking resources, and lowering the threshold. The first 2 categories contain barriers and facilitators, whereas the third category contains only facilitators. The categories lead to the more latent theme *Perceiving enough control to enable action*, meaning that it seems that perceiving the *right* amount of control is essential to maintain or increase the level of PA when using an eHealth tool among patients with COPD. However, the right amount of control seemed to depend on the individual (and context) in question.

**Conclusions:**

The core category indicates that a need for a certain sense of control was interpreted as necessary for increasing the PA level as well as for using an eHealth tool to help increase the PA level. The eHealth tool seemed to strengthen or weaken the perception of control by either providing support or by being too demanding on the user. Perceptions varied depending on other environmental factors. The Fogg Behavior Model illustrated how motivational levels, ability levels, and functional triggers interact within our findings. Thus, this study provides further evidence for the importance of empowering the patients to boost their level of agency and their ability to improve PA levels.

**International Registered Report Identifier (IRRID):**

RR2-10.1136/bmjopen-2019-030788

## Introduction

### Background

Among people with chronic obstructive pulmonary disease (COPD), low level of daily physical activity (PA) is the main risk factor for developing cardiovascular, metabolic, and musculoskeletal comorbidities [1,2]. It is also independently associated with poor clinical and prognostic outcomes, including an increased risk of hospitalization and mortality [2-5]. Notably, PA is markedly lower in patients with COPD, independent of disease severity [1,6,7]. Among people with a recent COPD diagnosis, a mild degree of airflow obstruction, and low symptom burden, the degree of PA is reduced by >30% compared with healthy older individuals [8].

Evidence suggests that people with COPD avoid participation in PA owing to the perception of breathlessness, leading to a vicious circle of muscle deconditioning, further compromising their capacity to engage in PA [3,9-12]. However, improving the level of PA in people with COPD is complex, as PA behavior itself is complex and multifaceted, including, for example, personal, physiological, and psychological elements as well as social and environmental factors [1,13,14]. Current strategies used to improve PA levels include, but are not limited to, exercise training, nutritional interventions, pharmacological treatments, and behavioral approaches that are used either in isolation or in combination depending on what is thought to be the limiting factor for the specific individual with COPD [3]. Notably, despite the wide range of available methods, their effect on PA in patients with COPD is limited, which might be attributed to an incomplete understanding of the determinants of PA behavior in the COPD population [12,15]. Although a variety of determinants of PA have been identified, including, but not limited to, exercise capacity, previous exacerbations, dyspnea, quadriceps dysfunction, and hyperinflation [11,15-17], the available information does not clearly answer the determinants of PA in patients with COPD. This is mostly because the available research has been cross-sectional [15]. Nevertheless, in a recent longitudinal study [12], determinants of PA after completing a pulmonary rehabilitation program were investigated in 350 patients with COPD. However, despite including a wide range of potentially relevant determinants such as movement-related basic abilities and skills (eg, meters walked on the 6-minute walk test), body- and PA-related basic knowledge (eg, knowledge on the benefits of PA), and personal dispositions and motivational characteristics (eg, self-efficacy), the best predictive model could explain roughly 30% to 40% of the variation in PA, thus suggesting that other potentially relevant determinants exist, warranting further investigation.

Interventions based on behavior change constructs, with long-term follow-up of PA after program completion, are expressly warranted [3,12]. We previously found that people with COPD who were given access to a web-based eHealth tool for 3 months were >4 times more likely to improve subjective PA at a 3-month follow-up than those not using the web-based eHealth tool [18]. They were also 2.7 times more likely to have improved their self-assessed PA level at 12 months (after program completion), although the latter was not statistically significant (*P*=.07) [18]. Importantly, we found that perceived competence of patients with COPD in performing PA was moderately to strongly correlated with the amount of self-reported PA and moderately correlated with the use of PA as a strategy to manage their disease across all time points, thus suggesting a link between the competence of PA and the amount of PA performed, in which a greater understanding of how to be active was associated with being more active [18]. In line with these findings, Carl et al [12] recently found that PA-specific self-control, self-efficacy, and emotional attitude (all 3 described as forms of self-regulation competence) toward PA were predictive factors of long-term PA behavior among people with COPD.

Similarly, among healthy individuals [19], the ability of individuals to steer PA toward positive affective reactions and subjective well-being enhances the probability of successful long-term adherence to regular PA. However, a model that integrates the functional, disease-related aspects and important psychological constructs to better understand the enablers and barriers of PA behavior in people with COPD is currently missing [12]. Furthermore, although electronic health solutions such as web-based support or websites have shown positive effects on PA in people with COPD [18,20], the results are inconclusive [21,22], and it is still unclear how eHealth solutions might be used to support positive changes in PA behavior in people with COPD [3,22]. In addition, availability and willingness to use eHealth tools have been found to be high in people with COPD in Sweden [23].

### Objective

Therefore, this study aimed to explore perceptions of people with COPD of changing their PA behavior using an eHealth tool.

## Methods

### Study Design

This study is part of a pragmatic randomized controlled trial (RCT), with in-depth interviews between the 3- and 12-month follow-ups [24]. This study is in line with the Consolidated Criteria for Reporting Qualitative research guidelines [25]. Individuals with a COPD diagnosis treated in primary health care were eligible for the RCT (registered at ClinicalTrials.gov; identifier: NCT03746873).

The methodology used was constructivist grounded theory (GT), following Charmaz’s [26] writing and description of the researcher’s part in the research process. For example, this means adhering to the assumption that previous knowledge and experience with researchers will influence choices when investigating, interviewing, analyzing, and presenting research [26].

### Ethics Approval

Ethics approval was provided by the Regional Ethical Board of Umeå University, Umeå, Sweden (Dnr: 2018-274-31 plus amendment Dnr: 2019-05572). Written informed consent was obtained from each participant before enrollment in the subsequent elective in-depth interview study.

### Setting and Sample

Purposive sampling included participants from the RCT intervention group who agreed to participate in the in-depth interviews. Primarily, SM, TS, and AN performed the sampling. The selection of participants was based and stratified based on 5 factors: use of the eHealth tool, defined as how many times the patient had reported visiting the COPD Web after receiving a prompt (SMS text message and email; low, 0%-25% of the times after receiving a prompt; medium, >25%-50%; or high, >50%); effect on PA (increase, no change, or decrease); geographical location (urban or rural, and north, middle or south); time of the year (to account for different seasons); and included both women and men. Later, theoretical sampling was used to confirm or disconfirm our findings [26]. For the theoretical sampling, the interviewed person was chosen according to whether they seemed to have information regarding the specified questions.

The purposive sampling led to 13 (n=7, 54% women) participants aged between 49 and 84 years and living in 8 municipalities in Middle and Northern Sweden. At the 3-month follow-up, 46% (6/13) of participants had increased PA levels by at least 600 steps per day, 23% (3/13) had an unchanged PA level (+ <600 or − <600 steps per day), and 31% (4/13) had decreased PA levels by at least 600 steps per day. The theoretical sampling included 3 (n=2, 67% women) participants aged between 69 and 75 years and living in 3 different municipalities in Middle and Northern Sweden.

A practical approach was chosen for the interviews. We preferred face-to-face interviews when participants lived near enough to reach in a day by bus or train and telephone interviews when participants lived further away. However, by the fourth interview, COVID-19 restrictions were imposed, and interviews were conducted only via telephone. Therefore, the 2 first interviews were conducted in person at a place that the participants chose (both times in their own home), and the rest of the interviews were conducted via telephone. All interviews were recorded using a Dictaphone.

### The eHealth Tool

**Figure 1:**
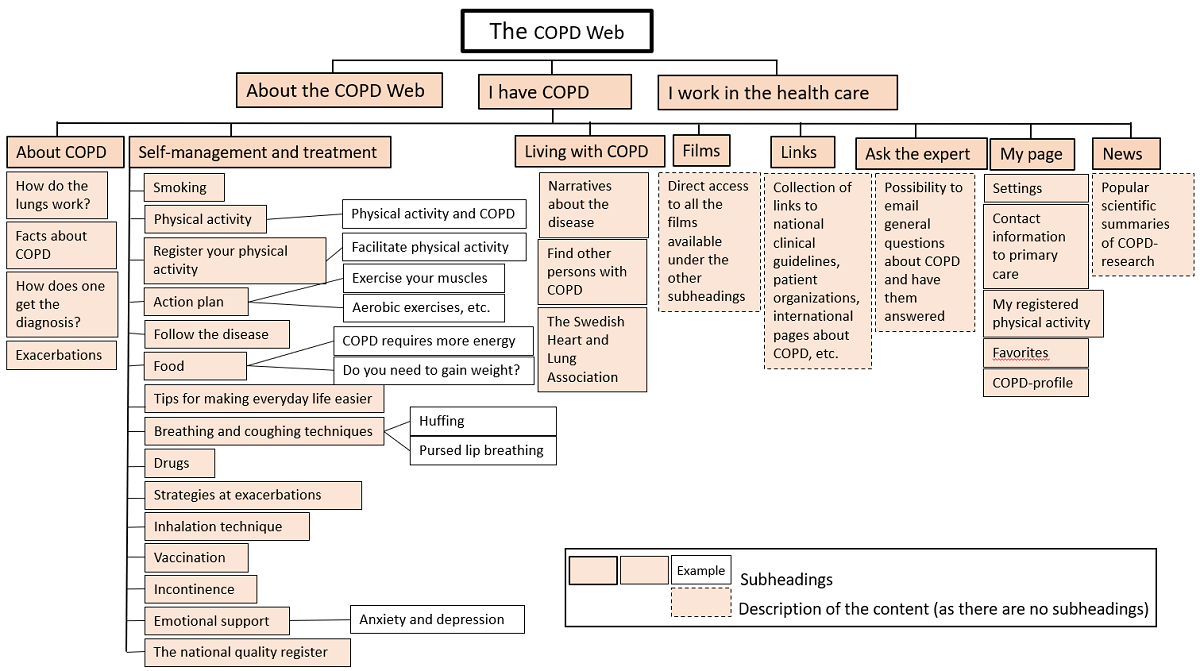
A website map of the COPD Web showing the section “I have COPD.” COPD: chronic obstructive pulmonary disease.

The COPD Web is a web-based eHealth tool developed for people with COPD, their relatives, health care professionals, and researchers using a standardized cocreation strategy. The development and design of the COPD Web and the experience of using the COPD Web among people with COPD have been extensively described elsewhere [18,27-29]. However, the section on PA within the COPD Web was explicitly targeted in this study (Figure 1). It includes a mix of images, written information, videos, and helpful links meant to support PA behavior by informing about COPD and health-improving activities and strategies, such as breathing techniques, advice on lessening the strain from everyday life tasks, PA, and exercise, as well as noticing the symptoms of exacerbations (Figure 1). On the COPD Web, it is also possible for the user to register their PA (number of steps per day), and to that end, all participants were given a step counter to use freely [24].

In addition, the participants received weekly prompts (SMS text message and email) [24]. The prompts included targeted information, referral links to the COPD Web, and a reminder to register counted steps to improve adherence to the intervention as previously used, targeting health behaviors, including PA in people with COPD [30].

### Process of Data Generation

Participants were contacted via telephone after their 3-month follow-up to reaffirm their interview stance. All except one still agreed to the interview. The reason for declining participation was not declared. Participants chose the preferred time, date, and location for the interview. They were asked to set aside 1 hour for the interview to minimize the risk of anyone feeling rushed.

Sociodemographic data, including age, sex, and geographic location in Sweden, were recorded. In addition, the participants’ PA level (defined as the difference between the mean number of steps at baseline before inclusion and at their 3-month follow-up) and self-rated compliance to the prompts (ie, how many times they had visited the COPD Web after receiving a prompt) were also gathered. Potentially eligible participants were recruited through primary health care units in different county councils in Sweden and advertisements in local and national newspapers.

Data were collected through individual semistructured qualitative interviews, and an interview guide (Multimedia Appendix 1) was used as the framework for the interviews. Questions or the interview guide were not provided beforehand to the participants. The interview guide consisted of the following main sections: action or agency, information seeking, expectations, health, lifestyle change processes, and technique and IT. The interview questions were asked in an order that best suited the conversation. All main areas were touched upon during the interviews. The duration of the interviews varied from 41 to 103 minutes.

While either SM (2 interviews) or a professional third-party transcriber (11 interviews) transcribed verbatim audio recordings, they were also revisited together with the field notes by SM to get to know the material and use the knowledge in the subsequent interviews. After receiving transcriptions from the transcriber, SM proofread the material. The transcripts were not shared with the participants for any feedback. At this stage, memoing was initiated. The memoing continued throughout the analytic process to verbalize and aid in the thought process. Initial coding was performed segment by segment, followed by a low abstraction level clustering used to highlight patterns and make the material easier to oversee. Subsequently, a mix of an axial and a conceptual coding phase started, and some rearranging was made to create a cluster order. These processes were iterative and were revisited throughout the analysis. Discussions among the researchers in the group were ongoing throughout the process, from the development of the interview guide to the final results. During the theoretical sampling phase, all 3 interviews were recorded, transcribed, and analyzed by SM in the same manner as before. These interviews varied between 27 and 76 minutes depending on how much the participant had to say about the subjects in question. All participants were assigned a pseudonym for transcription proofreading (used when quoting in the *Results* section).

### Research Team and Reflexivity

Interviewer SM (PhD student, certified physiotherapist, MSc in sociology, female, 34 years) conducted the interviews separately and had, before this study, performed >20 interviews (plus 2 years working with in-hospital patients). No previous relationships were established between the interviewer and the participants before the interview. However, the participants had some contact with TS during the recruitment phase and follow-up measurements before the interviews. All interviews began with a brief introduction to the interviewer’s background and the intention of the interview. To create an open, safe, and welcoming climate, the participants were encouraged to feel free to ask questions, omit answers, halt the interview, or ask for clarifications whenever (and without being questioned) throughout the conversation. All authors are certified physiotherapists, wherein AS and SM have previous experience and knowledge of the GT method.

In most of the interviews, only SM and participants were present, while some had their spouse in the vicinity during the interview (or part of the interview). Audio recordings and short field notes were used. Two immediate callbacks were made during the interviews (to clarify previously given information and ask a few additional questions). One participant called the researcher back with further details soon after the interview. All participants provided verbal consent to be contacted again if needed. After each interview, SM read and listened to the material and wrote additional field notes. The participants were not engaged in providing feedback on the findings. Topics of interest raised during interviews could be added in the following interviews.

### Analysis

Data were analyzed using constructivist GT because this method is appropriate when the goal is to study how and why participants make specific actions or choices in a particular situation [26]. In addition, interview transcriptions were coded primarily by SM, in collaboration with AN and AS, and software MAXQDA 2020 (VERBI Software GmbH) was used to facilitate data management during the analysis process. Finally, all categories and themes were constructed inductively from the data.

## Results

### Overview

The analysis resulted in the theme *Perceiving enough control to enable action.* The main categories *Welcoming or not welcoming action* and *Having or lacking resources* express both facilitators and barriers, whereas the last main category, *Lowering the threshold* only expresses facilitators within the setting (Textbox 1). The categories lead to the more latent theme *Perceiving enough control to enable action*, capturing the notion that having or perceiving a *right* amount of control seems essential to maintain or increase the level of PA when being diagnosed with COPD and using an eHealth tool. However, the right amount of control seemed to depend on the individual (and context) in question.

**Overview of the theme with categories and subcategories.**

**Perceiving enough control to enable action**

- Welcoming or not welcoming action:
  Feeling happy about physical activity (PA)
  Feeling happy about using an eHealth tool
  Lacking adequate incentives to act
- Having or lacking resources
  Managing the situation well
  Wanting for outside support to create ableness
  Identifying as incapable to act
- Lowering the threshold
  Using the environment or context as help to engage in PA

### Welcoming or Not Welcoming Action

Within this category, participants expressed facilitators and barriers related to doing or planning on doing PA. This is interpreted as a category in which factors related to motivation according to the Fogg Behavior Model (FBM) [31] are highlighted, for example, feeling positive effects from doing or in the prospect of doing the desired action—or, indeed, *not* feeling those positive effects or even negative dittos (representing the absence of motivational factors).

As facilitators, the participants expressed positive emotions and bodily sensations from PA. For example, feeling happy about oneself for completing a PA session, enjoying the environment or context in which one activate oneself (scenery and company), enjoying the activities themselves, reaching their PA goals, and having positive experiences from the past are mentioned in this category. In addition, setting goals for oneself, talking about wanting more PA, expressing an interest in PA, and describing a belief in what PA can do for them are voiced within this category:

I get a sense of wellbeing, movement and activity do me good.

Leena

Exercise is...well hell, it’s probably what’s kept me alive. It’s...I believe that! Totally!

Herbert

Continuing the description of facilitators, IT use was expressed as unproblematic and exciting. The eHealth tool was described as supportive, reassuring, instructive, useful, and able to help one feel heard, seen, and respected. The eHealth tool was interpreted as a motivational factor in increasing or maintaining PA levels:

I’ve been at it since I...I am pretty old...I’m 73, but I started using the computer you see...well, it was a long, long, long time ago.

Karina

I don’t always need to call the dang health center to ask what this or that is. I really don’t. Instead, I go and read here [on the COPD Web] a bit about these things.

Karina

As barriers, however, a lack of subjectively meaningful incentives to act was expressed, such as feeling that PA was tedious, that PA was not for them however beneficial for others, being satisfied with the current PA level, or for different reasons not having increased PA first in the priority list. In addition, a lack of incentives was heard when talking about using IT or performing information searches (including COPD-specific searches). Again, this indicates an insufficient motivation level for taking the desired action in this study:

But I think I’m doing relatively well anyway. I am. Because my COPD has gotten better rather than worse.

Angelica

The phone and the computer, that’s not what I prioritize when I get home.

Ingrid

### Having or Lacking Resources

Here, participants’ experiences were seen as expressing either a feeling of managing the situation well or not having adequate outside support to create ableness. This category highlighted ability-related factors, in line with FBM [31], as important for agency or action-taking. Resources found in the data include self-efficacy (the belief in one’s own capacity to act in the ways needed to reach a certain goal), social support, time, physical equipment, incentives or motivational factors, mental or physical ability, and helpful experiences, that is, anything that enhances your abilities to take a desirable action.

Facilitators here were participants disclosing feelings of being on top of their living conditions, for example, feeling able, hopeful, inspired, or having the time to do what they need or want. They also expressed such things as the ability to challenge themselves and the PA level, the ability to adapt to new situations and living conditions, and the feeling of having agency in one’s life:

I can’t grumble about how long I was a smoker, what’s done is done. But, to realize that I can actually make changes myself.

Mary (has been helped by COPD Web)

Facilitators were also expressed as, for example, feelings of acting out of duty, being a persistent person, having successfully managed a behavioral change, or improving or preserving your health status:

Things won’t get better, but I sure as hell won’t let them get worse.

Hector

As barriers, however, feelings of lacking resources to change and experiences of being socially unsupported were conveyed when talking about being the one that tended to others, getting too much or misguided “help,” or feeling ignored or not heard:

But then there was this with my husband’s illness and everything, many...yes, a lot of things like that on my mind. And so, I cut back on all that a bit.

Mary

The last two years, in the fall I’ve always gone to the doctor and complained that I can’t breathe...and that I can’t do what I used to do. Then they’ve done tests and said that it’s so little it’s hardly noticeable, they say.

George

Other contextual factors around the participants could also be insufficient in various ways, interpreted as not getting or having what you need, for example, work or pension could restrict the amount of spare time or PA opportunities, or technical gadgets or services could be malfunctioning or unavailable at times:

...has become less I guess since I retired...then you had to get to work and...I cycled there...And then...you were of course active...in your work, as it were...you were on the move all the time, as it were, in the handicraft classroom...and you walked a lot. You were responsible during recess, and you were outside...So, when all that ended, naturally a lot of that activity disappeared. Or all of that kind of activity, as it were.

Lars

The problem for me when I joined the COPD Web was that our computer, the one I used, it broke, just around that time.

Herbert

Participants could also be missing something to get going with their PA or use the eHealth tool. For example, experiencing that the eHealth tool contains the information they already are familiar with (making it feel uninteresting or not helpful), feeling like you need a certain bit of exercise equipment, or feeling inhibited instead of helped by some medication prescribed to you:

[talking to herself] Now, now, let’s go to the basement and switch on the computer and do these...oh no, but I don’t have one of those rubber bands.

Mary

There hasn’t been any aha moment like...oh God, I have to do that! Wow! That’s great! There hasn’t been anything like that. Instead, it’s been...yeah, yeah, yeah, fine, but I’m [already] doing that.

Angelica

And then [the doctor] said that your heart is beating too fast. I had a resting heart rate of 110 and it’s supposed to be 70...what’s to say 110 isn’t normal for me...he couldn’t really answer that of course, but I still got these tablets to lower my heart rate...And then I noticed that I had even less energy...And I don’t dare to just throw them away either.

Hector

In addition, the feeling of distrusting or not being a confident user of the internet or a computer was regarded as a barrier, as it was not interpreted as helpful in the study context.

In addition, feelings of being unable to increase PA were expressed in the form of being affected by shame, identifying as lazy, feeling overwhelmed or losing momentum (which could increase the threshold for doing PA), feeling blocked or hindered by fear, experiencing hopelessness, struggling to adapt to new situations, being under pressure, or feeling fatigued:

No, but, I...I don’t have the energy, I can’t get any air, you know, I wheeze, so when I meet people, they look at me, and I cough and get breathless when I try to walk. And when things get like that...your motivation just disappears...so yes, I’ve given up. It’s that simple.

Herbert

I guess I’m a bit lazy. I don’t go out running, let’s put it that way.

Anna

Finally, participants could express feeling incapable of increasing their PA level because of feeling too old, injury, or illness (including COPD symptoms), they voiced experiences of worsening symptoms because of some kind of weather, or felt incapable of executing PA although they tried to make their setting beneficial for it:

I don’t know how to get past the threshold, I try and I try.

Herbert

### Lowering the Threshold

This main category is built on the subcategory *using the environment or context to help engage in PA*, wherein experiences of feeling supported, being pushed into action, and being motivated by a lower PA threshold are voiced. This category is interpreted as highlighting trigger-related factors according to the FBM [31].

Participants expressed getting support from exercise groups, health care, the eHealth tool, comparing themselves with, or competing with, others, and walking the dog:

I had pretty good awareness already, but for me it felt like it [the COPD Web/the study] could be a form of support to get started and to continue being active and doing these movements, which are good for me.

Leena

The group pressure as well, because if [training partner in a wheelchair] does it, I sure as hell should be able to do it.

Hector

They also conveyed how things, people, or events could push them into action, such as the reminder sent from the eHealth tool, another bodily ailment (other than COPD), feedback from step counting, and fear:

A reminder, actually. A reminder. And these text messages, when they come...A reminder of your own situation and what you can actually do about it yourself. So that...yes...It’s motivated...it’s motivated me a lot, I think. I honestly think so.

Mary

[strength training...what was it that got you started with that?] It was the tension headaches...And I’d prefer to avoid taking painkillers...They’re not so good for your stomach and that.

George

The diagnosis scared me. I think that it’s an unpleasant diagnosis, but I’ve also realized that if I’m active and do what I can, I can keep it on the level I’m at now.

Leena

Participants also expressed ways to, or things that could, simplify action, for example, doing housework or having a hobby that was physically demanding, doing an activity that was natural to the current season, or having a routine to follow:

I know that if I can just get started the first time and re-make it a routine, then it’ll work again.

Angelica

In the countryside, I’m also active in the woods and pick mushrooms and berries and the like.

Lars

Just clearing the snow takes a hell of a lot of work, you know.

Herbert

## Discussion

### Principal Findings

This study aimed to explore the perceptions of increasing PA behavior when using a web-based tool among people with COPD. The main result was the core category *Perceiving enough control to enable action*, wherein the participants’ perceptions were interpreted to express a need for a certain sense of control over the environment and body to increase their PA level. Furthermore, the web-based tool was interpreted not only to strengthen but also weaken the feeling of control by either, for example, giving access to coping strategies or, for example, demanding too much from the user. Thus, users of an eHealth tool, such as the COPD Web, need to be susceptible to, and capable of, using these tools to benefit from them.

### Interpretation of Findings

Our findings support the notion that access to an eHealth tool should be considered for those with COPD who are ready for this type of intervention. It might provide additional practical, emotional, and psychological help when aspiring to increase one’s PA levels. This contradicts an earlier study by Robinson et al [32], in which the authors found that an internet-mediated intervention was significantly beneficial for people with COPD, who had low baseline self-efficacy, to increase their PA levels. Perhaps this is because of the layout of the intervention or something that makes their software more accessible to the patient group. Regarding the layout of the intervention, the authors mention the intervention including social support, which is something that our intervention did not include, but that we found motivating for our patient group.

Our findings also support the findings of previous studies in which eHealth solutions and control, or patient empowerment, have been seen as helping factors with regard to different disease management, including PA [33-35]. A higher sense of control was associated with an increased PA level, life satisfaction, and fewer perceived constraints [33]. During a 4-year follow-up, individuals with an increased sense of control had reduced their risk of mortality, not to mention reduced risk of lung disease [33]. Another link to be highlighted here is that a higher sense of control was linked to reduced levels of loneliness and a higher tendency to interact with friends, which according to our participants is a helping factor for increasing their PA level [33]. That is, a social component connected to the PA level was expressed to make one feel more motivated to engage in that activity. According to our study participants, this was also mentioned as a trigger that helped push one into action.

Furthermore, according to a study by Disler et al [34], people with COPD could enhance their sense of empowerment by using tele- and web-based interfaces where they had access to, for example, self-help, support on medical decisions, and increased knowledge of their condition, which made them more confident. This also corresponds with the statements of our study participants, who expressed feeling more confident about when they should seek professional help (and avoid waiting too long), among other things.

Similar to our findings, other studies support the notion that eHealth may be a helpful tool to enhance PA in people with COPD [29,36,37]. For example, in a similar way as Vorrink et al [36] expressed that eHealth tools could be used as a tool to provide patients with insight into their current PA level and that the eHealth tool supported them in increasing their PA, our patients with COPD expressed experiences of feeling supported, being pushed into action, and being motivated when using the eHealth tool.

Moreover, as our data seemed to exhibit the same crucial behavior change factors as the FBM [31] reports, the model was used to further interpret our findings. For example, similar to the FBM, our data interpretation argued for the need for motivators, abilities, and triggers in certain measures to enforce a PA behavior change. Regarding the latter, triggers in the form of prompts have previously been found to enable enhanced effectiveness on limited contact interventions targeting health behaviors including PA [38-40] and proved to be useful also in people with COPD [30]. Notably, not all 3 factors have to exist in equal measure. However, they need to complement each other in such a way that the behavior in question is or becomes a realistic and prioritized option for the individual. For example, if a person with COPD has a sufficiently high motivation to exercise, then the ability level to engage in said exercise might matter less than if a person has a lower motivational level [31]. Our data also indicated that an eHealth tool could help people feel more comfortable managing their COPD, symptoms, or PA. Sometimes the help could be that one was not afraid to tell close ones about the disease, that one could teach others about the condition, or that one understood one’s own body better. Help could also be that the tool supported previous findings [29], all of which indicated that the eHealth tool provided patients with COPD with at least some of the tools necessary to manage their condition [41].

### Strengths and Limitations

A possible strength of this study was that the interviewer did not participate in developing the eHealth tool, presumably providing the interviewer with a greater possibility to help facilitate for the participants to be honest and descriptive about their thoughts, reducing the risk of bias. Furthermore, 4 out of 5 authors had previous in-depth knowledge of COPD, 4 out of 5 authors had previously worked with qualitative research methods, and 2 had previously worked with GT. Triangulations of data and theory and bouts of peer debriefings were made throughout the research process to enhance trustworthiness [42]. Moreover, efforts were made continuously to even out the power differences between researcher and participant by giving the participants as much manifest power as possible, for example, encouraging the participants to choose the time and place for the interview and to ask the interviewer questions or to omit answers if needed [26].

The subjectivity of the interviewer and authors is mainly considered a strength of the constructivist GT method, as the researchers’ previous knowledge will impact what questions are asked, or what interpretations are made. Within the constructivist GT method, we assume that there are several truths and experiences (closely linked to social constructivism). The researcher plays a self-evident role in data gathering, analysis, and presentation. It is one of the premises for doing this work and does not disrupt it [26].

### Future Implications

Knowing how to empower the patients and give them more robust feelings of control is essential in all person-centered care [33]. In this study, we found further evidence of the importance of perceiving control in boosting a patient’s agency or action. In this respect, eHealth tools seem to be a beneficial support system for those qualified to use them. These tools seem to be supportive in various ways, practically, mentally, and emotionally, thus increasing the feeling of control. However, as using an eHealth tool also weakened the feeling of control in some participants, by demanding too much from the user, these findings indicate that eHealth tools may not be suitable for all patients with COPD. To determine when and for whom eHealth tools could be used, the next step would ideally be to develop a screening tool for patients with COPD to guide who to (and not to) engage in eHealth. Previously, the level of health literacy has been associated with the use of eHealth tools among patients with COPD [29,43,44], and it might be that assessing health literacy could be a promising way to screen patients with COPD for whom eHealth tools might be feasible to use. Although the latter needs to be established in future studies, a large number of diverse health literacy screening tools are available and have been used in other populations [45,46]. Other potentially relevant determinants of eHealth use include, but are not limited to, the level of education, self-efficacy, expected performance and effort of the patient as well as the involvement of health care professionals, and time constraints [36,47,48].

### Conclusions

This study aimed to explore the perceptions of changing PA behavior when using a web-based tool. The main result was the core category, *Perceiving enough control to enable action*. The core category indicated that a need for some sense of control was interpreted as necessary for both increasing the PA level and using an eHealth tool to help improve the PA level. The eHealth tool seemed to strengthen or weaken the perception of control by either providing support or by being too demanding on the user. However, no user was solely “strengthened” or “weakened” by the tool. However, perceptions varied depending on other environmental factors, and the FBM was used to illustrate how motivational levels, ability levels, and functional triggers interact within our findings. These factors might then affect the change in PA level (or the use of an eHealth tool for changing the PA level), and according to our data, the feeling of control is essential for these factors. Thus, this study provides further evidence of the importance of empowering the patients to boost their level of agency and their ability to improve PA levels. In summary, an eHealth tool may either help or disrupt a patient with COPD effort to increase their PA level, depending on context, personality, and possibly other factors that we have not focused on in this study.

## Appendix

Multimedia Appendix 1 Image of the interview guide.

## Abbreviations

COPD: chronic obstructive pulmonary disease
FBM: Fogg Behavior Model
GT: grounded theory
PA: physical activity
RCT: randomized controlled trial

## References

[1] Watz H, Pitta F, Rochester CL, Garcia-Aymerich J, ZuWallack R, Troosters T, Vaes AW, Puhan MA, Jehn M, Polkey MI, Vogiatzis I, Clini EM, Toth M, Gimeno-Santos E, Waschki B, Esteban C, Hayot M, Casaburi R, Porszasz J, McAuley E, Singh SJ, Langer D, Wouters EF, Magnussen H, Spruit MA (2014). An official European Respiratory Society statement on physical activity in COPD. Eur Respir J.

[2] Waschki B, Kirsten A, Holz O, Müller KC, Meyer T, Watz H, Magnussen H (2011). Physical activity is the strongest predictor of all-cause mortality in patients with COPD: a prospective cohort study. Chest.

[3] Burge AT, Cox NS, Abramson MJ, Holland AE (2020). Interventions for promoting physical activity in people with chronic obstructive pulmonary disease (COPD). Cochrane Database Syst Rev.

[4] Vaes AW, Garcia-Aymerich J, Marott JL, Benet M, Groenen MT, Schnohr P, Franssen FM, Vestbo J, Wouters EF, Lange P, Spruit MA (2014). Changes in physical activity and all-cause mortality in COPD. Eur Respir J.

[5] Van Remoortel H, Hornikx M, Langer D, Burtin C, Everaerts S, Verhamme P, Boonen S, Gosselink R, Decramer M, Troosters T, Janssens W (2014). Risk factors and comorbidities in the preclinical stages of chronic obstructive pulmonary disease. Am J Respir Crit Care Med.

[6] Vorrink SN, Kort HS, Troosters T, Lammers JW (2011). Level of daily physical activity in individuals with COPD compared with healthy controls. Respir Res.

[7] Geidl W, Carl J, Cassar S, Lehbert N, Mino E, Wittmann M, Wagner R, Schultz K, Pfeifer K (2019). Physical activity and sedentary behaviour patterns in 326 persons with COPD before starting a pulmonary rehabilitation: a cluster analysis. J Clin Med.

[8] Johnson-Warrington V, Harrison S, Mitchell K, Steiner M, Morgan M, Singh S (2014). Exercise capacity and physical activity in patients with COPD and healthy subjects classified as Medical Research Council dyspnea scale grade 2. J Cardiopulm Rehabil Prev.

[9] O'Donnell DE, Gebke KB (2014). Activity restriction in mild COPD: a challenging clinical problem. Int J Chron Obstruct Pulmon Dis.

[10] O'Donnell DE, Laveneziana P, Webb K, Neder JA (2014). Chronic obstructive pulmonary disease: clinical integrative physiology. Clin Chest Med.

[11] Frykholm E, Gephine S, Saey D, Lemson A, Klijn P, Bij de Vaate E, Maltais F, van Hees H, Nyberg A (2021). Isotonic quadriceps endurance is better associated with daily physical activity than quadriceps strength and power in COPD: an international multicentre cross-sectional trial. Sci Rep.

[12] Carl JA, Geidl W, Schuler M, Mino E, Lehbert N, Wittmann M, Schultz K, Pfeifer K (2021). Towards a better understanding of physical activity in people with COPD: predicting physical activity after pulmonary rehabilitation using an integrative competence model. Chron Respir Dis.

[13] Caspersen CJ, Powell KE, Christenson GM (1985). Physical activity, exercise, and physical fitness: definitions and distinctions for health-related research. Public Health Rep.

[14] Bauman AE, Reis RS, Sallis JF, Wells JC, Loos RJ, Martin BW, Lancet Physical Activity Series Working Group (2012). Correlates of physical activity: why are some people physically active and others not?. Lancet.

[15] Gimeno-Santos E, Frei A, Steurer-Stey C, de Batlle J, Rabinovich RA, Raste Y, Hopkinson NS, Polkey MI, van Remoortel H, Troosters T, Kulich K, Karlsson N, Puhan MA, Garcia-Aymerich J, PROactive consortium (2014). Determinants and outcomes of physical activity in patients with COPD: a systematic review. Thorax.

[16] Boutou AK, Raste Y, Demeyer H, Troosters T, Polkey MI, Vogiatzis I, Louvaris Z, Rabinovich RA, van der Molen T, Garcia-Aymerich J, Hopkinson NS (2019). Progression of physical inactivity in COPD patients: the effect of time and climate conditions - a multicenter prospective cohort study. Int J Chron Obstruct Pulmon Dis.

[17] Choi YA, Lee JS, Kim YH (2022). Association between physical activity and dynapenia in older adults with COPD: a nationwide survey. Sci Rep.

[18] Nyberg A, Tistad M, Wadell K (2019). Can the COPD web be used to promote self-management in patients with COPD in swedish primary care: a controlled pragmatic pilot trial with 3 month- and 12 month follow-up. Scand J Prim Health Care.

[19] Ekkekakis P, Brand R (2019). Affective responses to and automatic affective valuations of physical activity: fifty years of progress on the seminal question in exercise psychology. Psychol Sport Exerc.

[20] Lundell S, Holmner Å, Rehn B, Nyberg A, Wadell K (2015). Telehealthcare in COPD: a systematic review and meta-analysis on physical outcomes and dyspnea. Respir Med.

[21] Kuijpers W, Groen WG, Aaronson NK, van Harten WH (2013). A systematic review of web-based interventions for patient empowerment and physical activity in chronic diseases: relevance for cancer survivors. J Med Internet Res.

[22] Tsuchida Y, Vorrink SN (2019). The effect of eHealth interventions on physical activity in patients with chronic obstructive pulmonary disease: a mini review. J Cardiol Cardiovasc Sci.

[23] Sönnerfors P, Skavberg Roaldsen K, Ståhle A, Wadell K, Halvarsson A (2021). Access to, use, knowledge, and preferences for information technology and technical equipment among people with chronic obstructive pulmonary disease (COPD) in Sweden. A cross-sectional survey study. BMC Med Inform Decis Mak.

[24] Stenlund T, Nyberg A, Lundell S, Wadell K (2019). Web-based support for self-management strategies versus usual care for people with COPD in primary healthcare: a protocol for a randomised, 12-month, parallel-group pragmatic trial. BMJ Open.

[25] Tong A, Sainsbury P, Craig J (2007). Consolidated criteria for reporting qualitative research (COREQ): a 32-item checklist for interviews and focus groups. Int J Qual Health Care.

[26] Charmaz K (2014). Constructing Grounded Theory. 2nd edition.

[27] Nyberg A, Wadell K, Lindgren H, Tistad M (2017). Internet-based support for self-management strategies for people with COPD-protocol for a controlled pragmatic pilot trial of effectiveness and a process evaluation in primary healthcare. BMJ Open.

[28] Tistad M, Lundell S, Wiklund M, Nyberg A, Holmner Å, Wadell K (2018). Usefulness and relevance of an eHealth tool in supporting the self-management of chronic obstructive pulmonary disease: explorative qualitative study of a cocreative process. JMIR Hum Factors.

[29] Marklund S, Tistad M, Lundell S, Östrand L, Sörlin A, Boström C, Wadell K, Nyberg A (2021). Experiences and factors affecting usage of an eHealth tool for self-management among people with chronic obstructive pulmonary disease: qualitative study. J Med Internet Res.

[30] Demeyer H, Louvaris Z, Frei A, Rabinovich RA, de Jong C, Gimeno-Santos E, Loeckx M, Buttery SC, Rubio N, Van der Molen T, Hopkinson NS, Vogiatzis I, Puhan MA, Garcia-Aymerich J, Polkey MI, Troosters T, Mr Papp PROactive study group and the PROactive consortium (2017). Physical activity is increased by a 12-week semiautomated telecoaching programme in patients with COPD: a multicentre randomised controlled trial. Thorax.

[31] Fogg BJ (2019). Fogg Behavior Model.

[32] Robinson SA, Shimada SL, Quigley KS, Moy ML (2019). A web-based physical activity intervention benefits persons with low self-efficacy in COPD: results from a randomized controlled trial. J Behav Med.

[33] Hong JH, Lachman ME, Charles ST, Chen Y, Wilson CL, Nakamura JS, VanderWeele TJ, Kim ES (2021). The positive influence of sense of control on physical, behavioral, and psychosocial health in older adults: an outcome-wide approach. Prev Med.

[34] Disler RT, Appleton J, Smith TA, Hodson M, Inglis SC, Donesky D, Davidson PM (2015). Empowerment in people with COPD. Patient Intell.

[35] Irizarry T, Shoemake J, Nilsen ML, Czaja S, Beach S, DeVito Dabbs A (2017). Patient portals as a tool for health care engagement: a mixed-method study of older adults with varying levels of health literacy and prior patient portal use. J Med Internet Res.

[36] Vorrink S, Huisman C, Kort H, Troosters T, Lammers JW (2017). Perceptions of patients with chronic obstructive pulmonary disease and their physiotherapists regarding the use of an eHealth intervention. JMIR Hum Factors.

[37] Vorrink SN, Kort HS, Troosters T, Lammers JW (2016). A mobile phone app to stimulate daily physical activity in patients with chronic obstructive pulmonary disease: development, feasibility, and pilot studies. JMIR Mhealth Uhealth.

[38] Fry JP, Neff RA (2009). Periodic prompts and reminders in health promotion and health behavior interventions: systematic review. J Med Internet Res.

[39] Alkhaldi G, Hamilton FL, Lau R, Webster R, Michie S, Murray E (2016). The effectiveness of prompts to promote engagement with digital interventions: a systematic review. J Med Internet Res.

[40] Larouche ML, Mullane SL, Toledo MJ, Pereira MA, Huberty JL, Ainsworth BE, Buman MP (2018). Using point-of-choice prompts to reduce sedentary behavior in sit-stand workstation users. Front Public Health.

[41] Bourbeau J, Nault D, Dang-Tan T (2004). Self-management and behaviour modification in COPD. Patient Educ Couns.

[42] Sikolia D, Biros D, Mason M, Weiser M (2013). Trustworthiness of grounded theory methodology research in information systems. Proceedings of the 8th Midwest Association for Information Systems Conference.

[43] Stellefson M, Paige SR, Alber JM, Chaney BH, Chaney D, Apperson A, Mohan A (2019). Association between health literacy, electronic health literacy, disease-specific knowledge, and health-related quality of life among adults with chronic obstructive pulmonary disease: cross-sectional study. J Med Internet Res.

[44] Stellefson ML, Shuster JJ, Chaney BH, Paige SR, Alber JM, Chaney JD, Sriram PS (2018). Web-based health information seeking and eHealth literacy among patients living with chronic obstructive pulmonary disease (COPD). Health Commun.

[45] Kim H, Xie B (2017). Health literacy in the eHealth era: a systematic review of the literature. Patient Educ Couns.

[46] Collins SA, Currie LM, Bakken S, Vawdrey DK, Stone PW (2012). Health literacy screening instruments for eHealth applications: a systematic review. J Biomed Inform.

[47] de Veer AJ, Peeters JM, Brabers AE, Schellevis FG, Rademakers JJ, Francke AL (2015). Determinants of the intention to use e-Health by community dwelling older people. BMC Health Serv Res.

[48] van Zelst CM, Kasteleyn MJ, van Noort EM, Rutten-van Molken MP, Braunstahl GJ, Chavannes NH, In 't Veen JC (2021). The impact of the involvement of a healthcare professional on the usage of an eHealth platform: a retrospective observational COPD study. Respir Res.

