# Supplementary figures and images for: To Act or Not to Act—a Sense of Control Is Important for People With Chronic Obstructive Pulmonary Disease to Increase Physical Activity: Grounded Theory Study
Source: JMIR Form Res. 2023 Feb 3;7:e39969. doi: 10.2196/39969 (PMC9938439; doi:10.2196/39969)

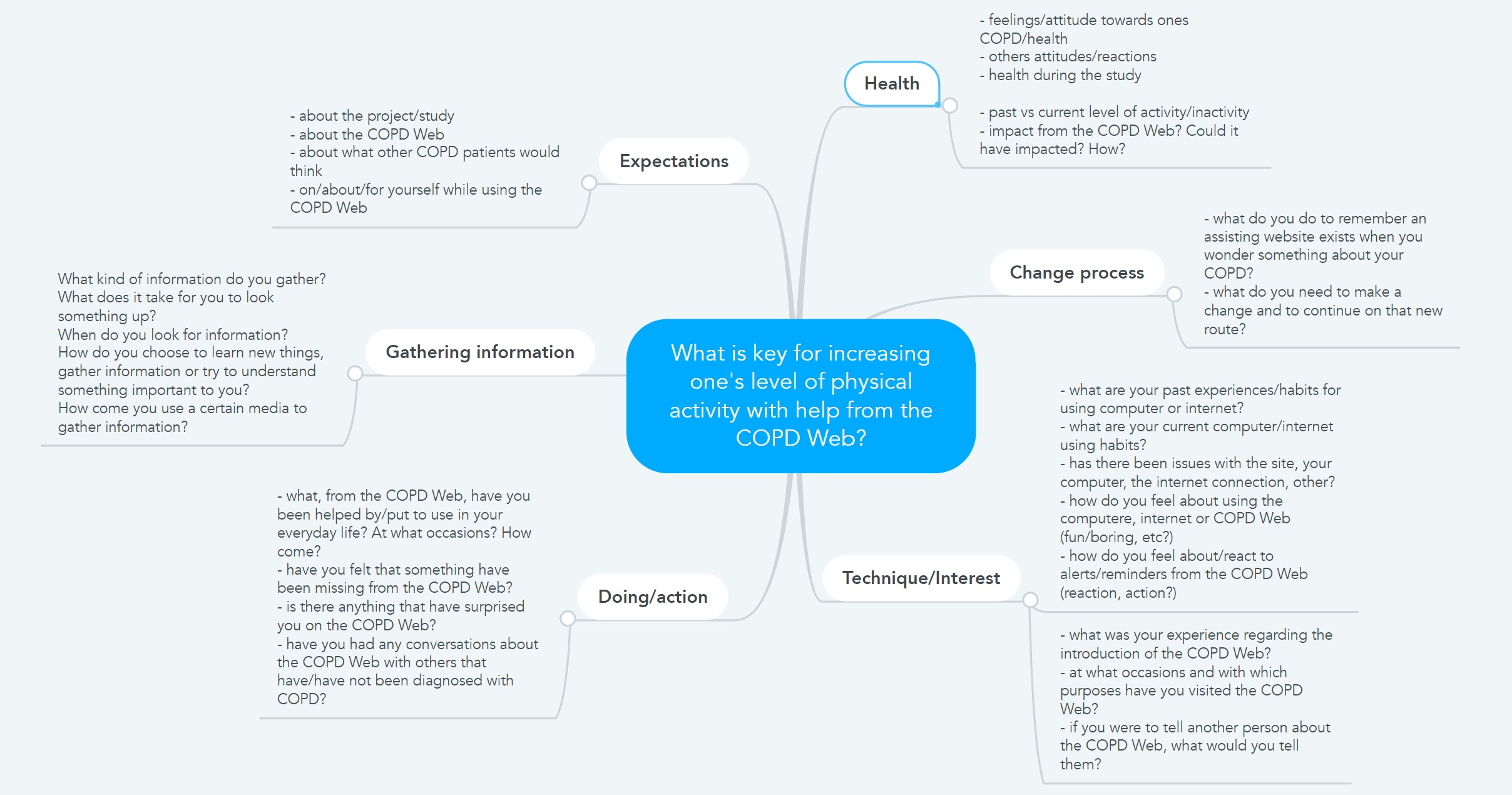

Supplement: Multimedia Appendix 1 [file formative_v7i1e39969_app1.png]
